# UHPLC-PAD Protocol for the Simultaneous Identification of Polyphenols and Bitter Acids: Organoleptic and Nutraceutical Fingerprinting of Bergamot-Flavored Craft Beer

**DOI:** 10.3390/foods13081149

**Published:** 2024-04-10

**Authors:** Sonia Carabetta, Rosa Di Sanzo, Pietro Andronaco, Francesco Canino, Tomas Branyik, Fabio Salafia, Salvatore Fuda, Adele Muscolo, Mariateresa Russo

**Affiliations:** 1 Food Chemistry, Safety and Sensoromic Laboratory (FoCuSS Lab), Department of Agriculture, Mediterranean University of Reggio Calabria, Via dell’Università, 25, 89124 Reggio Calabria, Italy; sonia.carabetta@unirc.it (S.C.); pietro.andronaco@unirc.it (P.A.); fabio.salafia@unirc.it (F.S.); salvatore.fuda@unirc.it (S.F.); mariateresa.russo@unirc.it (M.R.); 2 Laboratory of Pedology and Soil Ecology, Department of Agriculture, Mediterranean University of Reggio Calabria, Via dell’Università, 25, 89124 Reggio Calabria, Italy; francesco.canino@unirc.it (F.C.); amuscolo@unirc.it (A.M.); 3 Research Institute of Brewing and Malting, Lípová 511/15, 120 00 Prague, Czech Republic; branyik@beerresearch.cz

**Keywords:** phenols, bitter acids, beer, UHPLC-PAD, multivariate analysis

## Abstract

In this study, a UHPLC-PDA method for the simultaneous identification of polyphenols and bitter acids (alpha, beta, and isoalpha) in beer was developed. The resulting chemical profiles were leveraged to distinguish the characteristics of four (IPA, Lager, Blanche, ALE) bergamot-flavored beers, produced on a pilot-scale plant. In a streamlined 29 min analysis, thirty polyphenols and fourteen bitter acids were successfully identified under optimized separation conditions. Validation, encompassing parameters such as LOD (from 0.028 ppm for isorhamnetin to 0.106 for narirutin), LOQ (from 0.077 ppm for naringenin to 0.355 for narirutin), R^2^ (always more than 0.9992), repeatability (from 0.67% for tangeretin to 6.38% for myricetin), and reproducibility (from 0.99% for sinensetin to 6% for naringin), was conducted for polyphenol quantification using constructed calibration curves with seven levels. Exploring polyphenolic components as potential discriminators among different beer styles, a total of thirty-two polyphenolic compounds were identified and quantified, including characteristic bergamot peel polyphenols like neoeriocitrin (from 7.85 ppm for CBS2 to 11.95 ppm in CBS1); naringin (from 4.56 ppm for CBS4 to 10.96 in CBS1), and neohesperidin (from 5.93 in CBS3 to 15.95 for CBS2). The multivariate analysis provided additional insights into variations among specific beer styles, revealing discrepancies in the presence or relative concentrations of specific compounds linked to brewing ingredients and processes. This research enhances the fingerprinting of the chemistry governing beer quality through a straightforward and cost-effective analytical approach.

## 1. Introduction

Beer, a globally cherished alcoholic libation, occupies a distinguished status among the most favored beverages. Its origins date back approximately 6000–8000 years, when early renditions likely boasted a sweet, malty essence complemented by infusions of herbs, spices, or fruits—deviating significantly from the contemporary brew we recognize today [1].

Across epochs, an array of beer styles have unfolded, spanning from the crisp and invigorating pilsners to the robust and malty porters and the opaque, hop-laden Indian pale ales (IPAs). Despite the kaleidoscope of hues and flavor profiles, modern beer varieties derive their essence from the alcoholic fermentation of a must concocted with four primary raw components: malted and unmalted cereals, water, hops (*Humulus lupulus* L.), and yeast [2]. In 2018, global beer production peaked at nearly two billion hectolitres, as documented by statista.com. The surge in independent microbreweries, particularly in Italy’s Calabria region, has spurred a renaissance of experimentation with ingredients and brewing methodologies. This innovative wave has birthed a new era of beers—sophisticated, intricate, and bursting with flavor. As a result, this upsurge in a myriad of beer offerings has sparked increased curiosity and enthusiasm for speciality brews, emphasizing a growing need for accurate and dependable flavor profiling techniques.

The chemical makeup of beer, influencing its taste, aroma, and color, undergoes dynamic transformations based on the diversity of raw materials and production methods [3]. Delving into the flavors of beer at the chemical level proves to be a nuanced task, given the intricate nature of this beverage—a complex amalgamation of various elements such as carbohydrates, proteins, microbes, secondary metabolites, sulfur dioxide, and ethanol. The palate of beer is shaped by a myriad of compounds, with polyphenols playing a significant role, sourced 80% from cereals and 20% from hops [4].

Phenols, pivotal not only in defining beer’s taste but also in enhancing its long-term stability, play a crucial role in the fermentation product’s preservation. Notably, polyphenols like flavon-3-oils exhibit a natural ability to chelate metals within the solution. Simultaneously, various phenolic families act as inhibitors during oxidative processes, curbing the generation of diketones, sulfur compounds, aldehydes, and low molecular weight fatty acids—molecules implicated in the beer maturation process [5,6].

Another class of influential molecules shaping beer’s aroma, flavor, and shelf life is found in the bitter acids of hops. Concentrating in the resin of hops, these bitter acids come in two categories: α-acids or humulons (humulone, cohumulone, adhumulone as primary and prehumulone, posthumulone as secondary) and β-acids or lupulones (lupulone, colupulone, adlupulone as primary and prelupulon, postlupulon as secondary) [7,8,9].

During the boiling of the must and preceding the fermentation phase, these bitter acids undergo isomerization, a process influenced by various factors such as the quality, quantity, aging degree, and form (cone, pellet, or plug) of the hops. Additionally, the duration and temperature of boiling, pH, and the presence of bivalent cations in the must impact the isomerization yield [7,8,9]. Humulons, through isomerization, give rise to iso-α-acids or iso-humulons in cis and trans forms, exhibiting a heightened bitterness effect compared to their precursors. In contrast, lupolones remain unaltered during isomerization, forming cyclic and epoxy compounds [10].

Beers with elevated levels of bitter acids, such as Indian pale ale (IPA) and American pale ale (APA), are often perceived as less bitter by consumers compared to beers with a moderate bitter acid content, like Bitter and Strong Bitter. This intriguing phenomenon arises from the specific type of bitter acids present in beer and the balancing influence of phenols on the bitter taste [1,3,11].

Moreover, the bitter acids found in hops have garnered attention for their potential antiviral, antibacterial, and anti-inflammatory properties [12,13,14,15].

The polyphenolic profile of craft beers, primarily influenced by barley and hops, serves as a key indicator not only of nutritional and antioxidant quality but also of colloidal stability and the beer’s ability to interact with proteins. Importantly, this profile profoundly shapes sensory characteristics such as color, aroma, and flavor. Hence, it becomes imperative to establish a rapid, straightforward, and cost-effective method readily available in standard analysis laboratories to accurately trace the polyphenolic fingerprint of beers. Traditionally, total phenols and total bitter acids in beers are quantified using distinct spectrophotometric methods developed by the American Society of Brewing Chemists (ASBC) and European Brewery Convention (EBC) [3,16,17,18,19].

Chromatographic methods, designed for the identification and quantification of phenolic acids and flavonoids, have also been developed [4,20,21,22].

While some methods involve liquid–liquid extraction (LLE) with organic solvents [23,24], others opt for sample injection without treatment, capturing only a subset of phenols [25,26]. Few studies exist for the identification of different bitter acids in beers using chromatographic methods, and these typically employ liquid–liquid extraction (LLE) with organic solvents [27,28,29,30]. Only one published work addresses the qualitative identification of prenylflavonoids and bitter acids in beers [31].

This study aimed to develop and validate an ultra-high-performance liquid chromatography method coupled with a photodiode array detector (UHPLC-PDA) for the simultaneous analysis of diverse phenolic classes and the numerous bitter acids present in beers, employing a rapid and simple sample preparation. To the best of the authors’ knowledge, there is only one analytical method in the literature for simultaneous phenol and bitter acid identification in beers [32] using liquid chromatography-quadrupole time-of-flight mass spectrometry (LC-MS). Despite LC-MS’s high selectivity and specificity, UHPLC-PAD proves to be a more accessible choice for small laboratories, offering comprehensive assessment capabilities for specific chemical compounds. This makes UHPLC-PAD the preferred option for delving into the extensive chemical variations seen in the products produced by today’s craft brewing industry.

The polyphenolic composition of beer can undergo substantial changes due to the use of specific cereals, hops, and “characterizing foods” such as fruits, herbs, and juices employed in the production of flavored beers. Beers crafted with citrus juices, like those with bergamot or with grape must (Italian Grape Ale or IGA), exhibit phenols absent in traditional beers, originating from these “characterizing foods.” Employing advanced analytical techniques to explore characterizing profiles in the brewing industry holds the potential to facilitate the development of new flavors, bolster quality control procedures, and provide brewers with a deeper understanding of how ingredients and processes shape their final products. This not only expands the market appeal of beers but also offers a more profound insight into their chemical profiles.

This study aimed to conduct a simultaneous analysis of IAAs (Iso-α-Acids) and phenolic compounds in beer using a straightforward UHPLC-PAD procedure, leveraging the resulting chemical profiles to assess the ability to discriminate the polyphenolic profile and flavor of beers with different styles yet all flavored with bergamot.

## 2. Materials and Methods

Ultrapure water was obtained using a Milli-Q system (Millipore, Milan, Italy), formic acid and acetonitrile (ACN) with UHPLC grade of purity were purchased from Sigma-Aldrich (Milan, Italy), while the phenol standards (p-Coumaric acid, 4-Hydroxybenzoic acid, caffeic acid, ethylgallate, Ferulic acid, Kampferol, naringin, Protocatechuic acid, Rutin, Syrengin acid, vanillic acid, Hesperidin, sinensetin, Neodiosmin, neoeriocitrin, Hesperetin, (-)epicatechin, Eriocetrin, isorhamnetin, myricetin, neohesperidin, Diosmin, narirutin, Rhamnetin, tangeretin, Apigenin, chlorogenic acid, Nobiletin, naringenin) were purchased from Extrasynthese (Genay Cedex, France). The bitter acid standards (ICE-4, ICS-I4, ICS-R3, ICS-H2, and ICS-T3) were obtained from the American Society of Brewing Chemists (ASBC) (St. Paul, MN, USA). ICE-4 contains Cohumulone 10.98%, Colupulone 3.02%, Humulone/Adhumulone 31.60%, Hupulone/Adlupulone 13.52%, with total α-acids 42.58% and total β-acids 26.54%. ICS-I4 contains trans-Isocoumulone, trans-Isohumulone, and trans-Isoadhumulone with total trans-iso-α-acids 65.2%. ICS-R3 containing cis-ρ-Isocoumulones, cis-ρ-Isohumulones, and cis-ρ-Isoadhumulones with total cis-ρ-iso-α-acids 65%. ICS-H2 containing cis-Hexaidroisocoumuloni, cis-Hexaidroisoumuloni, and cis-Hexaidroisoadhumuloni with total cis-Hexahydro-iso-α-acids 65.7%. ICS-T3 contains cis-trans-Tetrahydroisocoumulones, cis-trans-Tetraidroisohumuloni, and cis-trans-Tetrahydroisoadhumulones with total cis-trans-Tetrahydro-iso-α-acids 99.4%. The bitter acid standards purchased are all those currently available on the market. For bitter unit: hydrochloric acid and isooctane were purchased from Sigma Aldrich. For total phenols: carboxymethyl cellulose, sodium salt, ethylenediaminetetraacetic acid, disodium salt dihydrate, ammonium iron (III) citrate, 25% ammonia solution were purchased from Sigma.

### 2.1. Sample Preparation and Analysis

The entire work and analysis were conducted in Calabria (South Italy) at FocussLab Mediterranea University of Reggio Calabria, in 2023. The beers have been created using different recipes, which are listed below along with the codes used for their identification:

❖ CBS1: water, hops, barley malt, and bergamot peels
❖ CBS2: water, hops, barley malt, wheat, and bergamot peels
❖ CBS3: water, hops, barley malt, oat flakes, honey, bergamot peels, black pepper, and coriander
❖ CBS4: water, hops, barley malt, rye, and bergamot

Barley malt, yeasts, hops, and bergamot were the same for the preparation of all three beers. The beers were differentiated only for cereals and flavoring. This was in order to verify the potential of the new protocols developed for the study of polyphenolic fingerprinting. All beer samples were placed in a refrigerator at 4 °C in the dark and then degassed by magnetic stirring (500 rpm) for 8 h. Subsequently, the beer samples were filtered through a 0.45 μm regenerated cell membrane filter (Aisino Corporation, London, UK) and analyzed immediately after degasification pretreatment to reduce experimental errors caused by temperature and instrument instability.

### 2.2. Bitter Unit and Total Phenols by UV-VIS

For the determination of the bitter unit, a standard method EBC was used (Analytica-EBC, Section 9 Beer, Method 9.6.) For the determination of total phenols, a standard method EBC was used (RIF Analytica-EBC, Section 9 Beer, Method 9.11) [33].

### 2.3. UHPLC-PDA Instrumentation

The analyses were carried out with a Shimadzu Nexera UHPLC-PDA system (Shimadzu, Kyoto, Japan), composed of a controller (CBM-20A), a degasser (DGU-20A5R), dual-plunger parallel-flow pumps (LC-30AD), an autosampler (SIL-30AC), a column oven (CTO-20AC), and a photodiode detector (SPD-M30A). LC data processing was performed with LC solution software (Version 5.71, Shimadzu, Kyoto, Japan).

### 2.4. UHPLC-PDA Condition

The analytical conditions used for the analyses were optimized to obtain the best chromatographic separation for the classes of molecules considered, phenols and bitter acids. Ten microliters of the degassed and microfiltered sample were injected without performing preliminary extraction procedures. The chromatographic separation was carried out with a Kinetex C18 column (50 mm × 3 mm × 1.7 μm d.p) and a Kinetex C18 pre-column; the columns are manufactured by Phenomenex (Torrance, CA, USA). The oven temperature was set at 40 °C, and a flow rate of 0.6 mL/min was used with mobile phases composed of water with 0.1% formic acid (*v*/*v*) (mobile phase A) and acetonitrile with 0.1% formic acid (*v*/*v*) (mobile phase B). The gradient used was as follows: 5 min with 1% B (isocratic mode), 15 min from 1% to 30% B, 3.5 min from 30% to 47% B (gradient mode for the separation of polyphenols), 30 s from 47% at 60% B, 2 min with 60% B (isocratic mode for the separation of bitter acids), washing the system with 100% B and reconditioning with 1% B. The photodiode detector was set with 8 nm divided width, 256 spectrum resolution, 40 Hz sampling rate, 40 °C cell temperature, and 190–450 nm analysis range.

### 2.5. Validation Method for Polyphenols

Seven concentration levels of the polyphenolic standards were prepared with methanol from a 1000 mg/L stock solution with a concentration range of 0.5–120 mg/L. Five analyses were performed for each concentration level with the UHPLC-PDA system under optimized chromatographic conditions. Seven-level calibration curves were constructed using the least squares method by obtaining the equations of the regression lines (Table S1). Mandel’s test confirmed the linearity of each calibration curve in the considered range. The limit of quantification (LOQ) and limit of detection (LOD) (Table S1) were calculated by multiplying the standard deviation (SD) of the lowest level of the calibration curve (*n* = 7) ten and three times, respectively, and dividing the result for the slope of the calibration curve. The repeatability and reproducibility values (Table S1) were expressed as percentage coefficient of variation (CV%) and calculated using the average of the areas of the lowest level of the calibration curve (*n* = 7) divided by the corresponding standard deviations. Finally, retention time, instrumental recovery, and percentage relative standard deviation (RSD%) were determined using the fourth level (*n* = 4) of each calibration curve (Table S2).

### 2.6. Matrix Effect

To assess potential interferences in compound quantification stemming from direct beer injection, we conducted a thorough evaluation of matrix effects (MEs). Introducing variability, we incorporated two phenols with distinct polarities, namely, 4-Hydroxybenzoic acid and tangeretin, as internal standards (IS) into both blonde beers, characterized by a simple polyphenolic composition, and dark beer, known for its complex polyphenolic makeup [2,3]. Matrix calibration curves were meticulously established at four levels, covering n = 1 and n = 7 of the solvent calibration curves, following the methodology outlined by Gosetti et al. [34]. ME was computed using the formulas outlined by Gosetti et al. and Trufelli et al. [34,35], leveraging the slopes of the calibration curves in both solvent and matrix.

### 2.7. Statistical Analysis

The data were analyzed using XLSTAT software (Version 2022.4.5, Addinsoft, Paris, France). All the data were subjected to analysis of variance (ANOVA). ANOVA was applied to the antioxidant profile. The means were separated using the Tukey test only when the F-test for treatments and interactions was significant at the *p* ≤ 0.05 probability level. Principal component analysis (PCA) was conducted using PCA-XLSTAT software version 2015.5 by Addinsoft, Paris, France, to assess datasets of antioxidant compounds, bitter units, and total phenols.

## 3. Results

**Figure 1:**
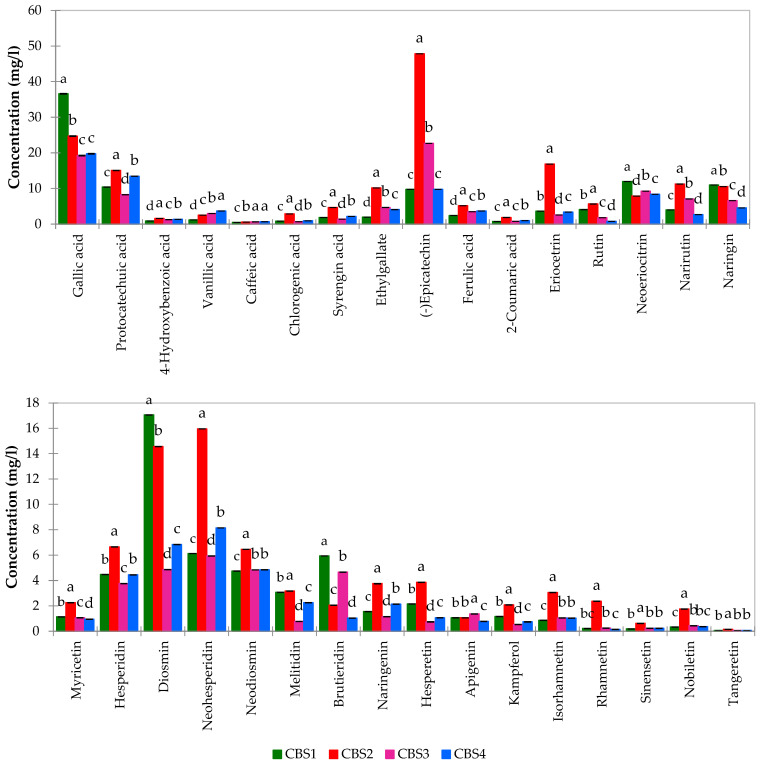
Polyphenols concentration (means between three replicates) of beer samples. Different lowercase letters (a–d) indicate significant differences (*p* < 0.05) between the samples.

In a streamlined 29 min analysis, thirty polyphenols and fourteen bitter acids were successfully identified under optimized separation conditions. Validation was conducted for polyphenol quantification using constructed calibration curves with seven levels. In Table S1, thirty polyphenol compounds were reported according to their optimization values. In particular, LOD, LOQ, R^2^, repeatability, and reproducibility were evaluated, and they were in the following ranges: LOD from 0.028 ppm for isorhamnetin to 0.106 for narirutin, LOQ from 0.077 ppm for naringenin to 0.355 for narirutin, R^2^ always more than 0.9992, repeatability from 0.67% for tangeretin to 6.38% for myricetin, and reproducibility from 0.99% for sinensetin to 6% for naringin. In Table S2, for the same polyphenols, retention time, instrumental recovery, and percentage relative standard deviation are reported. The recovery was very high, always above 90%, except for Diosmin which showed a value of 86,9%. Also, five bitter acid standards (ICE-4, ICS-I4, ICS-R3, ICS-H2, ICS-T3) were analyzed individually by the method described, and all compounds in the standards were separated to the best of instrumental capability. UHPLC system with sub-2 core–shell column allowed us to separate compounds with very similar structures such as cis–trans pairs (trans-tetrahydroiso Cohumulone/cis-tetrahydroiso Cohumulone and trans-tetrahydroiso Humulone/cis-tetrahydroiso Humulone). Some analytes coelute due to the very similar chemical structure such as trans-iso Adhumulone and cis-ρ-iso Adhumulone, while Adlupulone/Lupulone and Adhumulone/Humulone coelute because they are quite apolar, and a reverse phase isocratic elution does not allow their separation. These coelutions are reported in several publications [28,29,30] in Table S3, the compounds identified for each standard, and related coelutions have been reported. To complete the validation method, for tangeretin and 4-Hydroxybenzoic acid, the equation of calibration curves in solvent and in matrix were evaluated and, with matrix effect value (ME%), are reported in Table S4. Both the equation and ME were evaluated in dark and blonde beer showing a minor ME in the dark beer. Notably, the matrix effect observed for both types of beer falls into the suppression category (as indicated in Table S4) yet remains well within the acceptable range of 80% to 120% [36], affirming the robustness of our method in handling complex matrices. This extensive study on matrix effects (MEs) robustly affirms that the direct injection of beer, while not considering potential coelutions, does not significantly interfere with the quantification of polyphenols. This underscores the reliability of our method in maintaining accuracy amidst the complexity of beer matrices. Polyphenols and bitter acids were determined in beers as an application of the proposed method. The identification of the compounds in the beer samples was made considering the retention times, the profile of the UV absorption spectra, and the literature data (for Melitidin and Brutieridin quantified as naringenin, being typical compounds of bergamot). Total concentrations of polyphenols were compared to assess the relative phenolic content between beer styles. The two IPA beers exhibited higher concentrations of polyphenols, with an average amount of 238.91 mg/L for CBS2, enriched with wheat and bergamot peels, compared to 151.75 mg/L for CBS1 without wheat. The ALE beer (CBS4), enriched with rye and bergamot peels, demonstrated the lowest overall phenolic content among the analyzed beers. Notably, epicatechin and gallic acid emerged as the predominant phenolic compounds across all beer styles. In particular, CBS1 stood out for its elevated concentration of gallic acid, averaging 36.87 mg/L, while IPA CBS2 exhibited higher levels of (-) epicatechin, reaching an average value of 47.87 mg/L. This value significantly surpassed other IPA variants such as CBS1 (9.75 mg/L) and was notably higher than the ale (9.71 mg/L) and blanche (22.68 mg/L). The IPA (CBS2) beer displayed a pronounced expression of flavonoids, as evidenced by Figure 1.

In this particular beer variety, Eriocetrin, Diosmin, neohesperidin, narirutin, and naringin stood out as notably abundant compounds. The IPA style (CBS1 and CBS2) distinguished itself with a higher concentration of these compounds, with naringin, narirutin, and neohesperidin particularly prominent in wheat beer compared to other beer styles. Moreover, IPA (CBS2) exhibited the highest levels of gallic acid, Protocatechuic acid, and ethylgallate. CBS2 also showcased a heightened concentration of chlorogenic acid compared to other beers, suspected to have originated from the distinctive brewing ingredients. Significantly, previous studies have identified chlorogenic acid as a major phenolic acid present in oranges and other citrus fruits [32]. Total phenols and bitter units were also determined for these beers, being integral components of the broader quality control processes in the brewing industry aimed at maintaining consistency in taste and characteristics. bitter units (BUs) or International Bitterness Units (IBUs) serve as a metric for gauging the bitterness or hoppy flavor in beer. This value quantifies the amount of bitter compounds, primarily iso-alpha acids derived from hops in the beer. The measurement and control of both bitter units (BUs) and total phenols in beer are crucial for brewers to ensure the desired flavor profile and quality of their products. They play a pivotal role in discriminating between different styles of beer, contributing to both positive and negative characteristics in terms of flavor and aroma.

**Figure 2:**
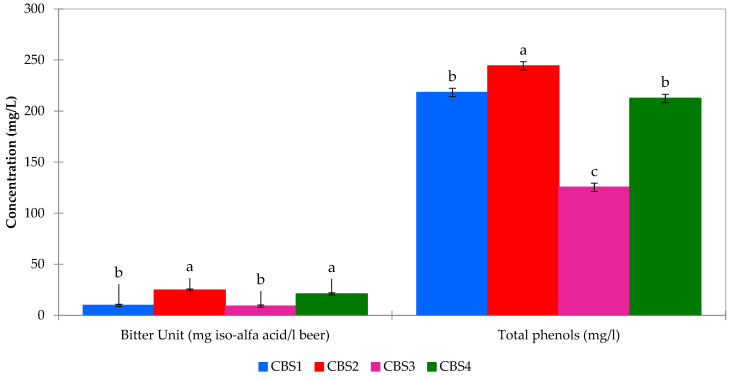
Bitter unit and total phenol content of beer samples. Different lowercase letters (a–c) indicate significant differences (*p* < 0.05) between the samples.

This comprehensive assessment remains essential for brewers to uphold the distinctive qualities of their beer varieties (Figure 2).

The evaluation of samples aimed to assess whether the style of beer could be effectively classified based on phenolic content.

## 4. Discussion

Phenolic acids, renowned for their antioxidant properties, play a pivotal role in mitigating the adverse effects of oxidative stress. In the evaluation of beer production and marketing quality, the polyphenolic composition emerges as a critical benchmark [8]. It is crucial to recognize that the type and quantity of phenolic compounds exert a profound influence on various facets of beer, encompassing taste, aroma, color, colloidal stability, foam retention, and shelf-life. Beer, a rich source of diverse phenolic compounds, is primarily categorized into phenolic acids, tannins, flavones, and flavonols [9]. Among these, phenols in alcoholic beers act as protective agents, shielding yeast from stress induced by high ethanol levels, akin to the role resveratrol plays in wine [13]. This highlights that phenolic compounds not only undergo changes during brewing but actively shape the brewing process.

**Figure 3:**
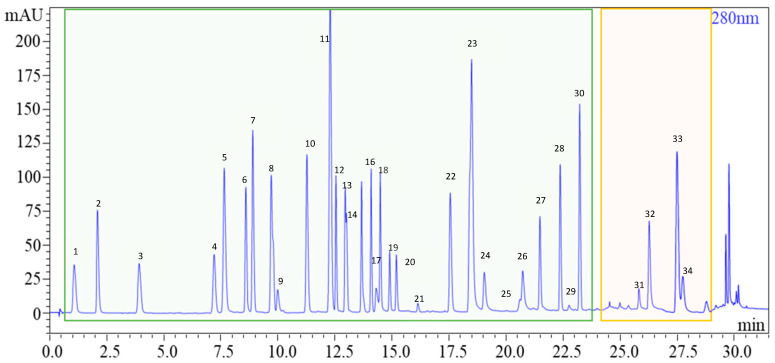
Chromatogram of the polyphenolic standards considered and of the ICE-4 standard containing α-β-acids.

This study harnessed advanced analytical techniques, such as UHPLC-PDA, to unveil the distinctive fingerprint of craft beers. We successfully developed and validated a swift, uncomplicated sample preparation method, enabling the simultaneous analysis of diverse phenolic classes and numerous bitter acids in beers. The method for simultaneous identification of polyphenols and bitter acids underwent refinement through meticulous optimization of various analytical parameters. This optimization process encompassed crucial aspects such as the selection of columns, acidifiers for the mobile phase, flow rate, and oven temperature. In the previous literature, C18 columns were a prevalent choice for chromatographically separating polyphenols [20,37,38,39] and bitter acids [16]. Our investigation revealed that the Kinetex C18 50 mm × 3 mm × 1.7 μm dp (particle size distribution) column outperformed the Kinetex C18 100 mm × 2.1 mm × 2.6 μm dp column, primarily attributable to the utilization of sub-2 core–shell particles as the stationary phase. Subsequently, two different acidifiers were scrutinized for mobile phase acidification. While formic acid is conventionally employed to enhance the chromatographic separation of polyphenols [20], phosphoric acid is preferred for bitter acid analysis [28]. Significantly, when both acids were used at a 0.1% *v*/*v* concentration, no substantial differences were observed, leading to the selection of formic acid as the optimal acidifier. Following this decision, parameters such as flow rate, oven temperature, and injection volume underwent meticulous fine-tuning for optimal performance. To further enhance chromatographic separation, we refined the mobile phase composition, implementing a gradient mode for polyphenols and an isocratic mode for bitter acids. This strategic adjustment allowed for the creation of a chromatogram neatly divided into two distinct sections: the green segment representing polyphenols and the orange segment representing bitter acids (see Figure 3). Table S5 presents the peak names and retention times of the compounds depicted in Figure 3. Our method achieved remarkable elution times, with approximately 24 min for polyphenols and 5 min for bitter acids. This represents a significant reduction in analysis times compared to existing liquid chromatography systems documented in the literature [4,20,21,22,23,24,25,26,27,28,29,40], underscoring the efficiency and speed of our approach.

Furthermore, our method, through direct injection without any sample pretreatment, even if utilizing a less sensitive technique compared to LC-MS/MS [41,42,43,44], ensured excellent results in terms of analytical instrument performance. Anderson et al. [32], for instance, reported the target profiling of beer styles by IAAs and phenolic compounds using LC-QTOF showing a LOQ (0.33 to 16.5 mg/L) and LOD (0.1 to 3 mg/L) higher than our method and only for ten compounds. Cortese et al. [43] provided an accurate and comprehensive quantification of phenolic compounds in craft beers showing LOD and LOQ values similar to our method, but they considered only 20 phenolic compounds utilizing a complex sample pretreatment and extraction procedure as Cheiran et al. [44] that, through a HPLC-ESI-MS/MS, found 57 phenolic compounds in craft beers. However, in our method, direct injection of the beer as it is, following simple filtration, was implemented.

**Figure 4:**
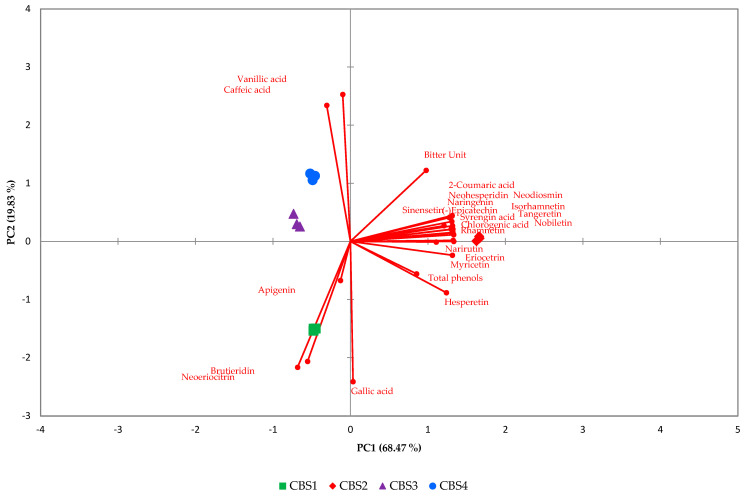
Bi-plot PCA of the four analyzed beers.

Leveraging the resulting chemical profiles, we meticulously evaluated our method’s efficacy in discriminating the polyphenolic profile of beers with different styles, all flavored with bergamot. Concerning the qualitative and quantitative analysis of polyphenols in craft beers, noteworthy differences surfaced. The principal component analysis (PCA) model successfully differentiated between the various beers (Figure 4). PC1, which accounts for 68.47% of the total variance. Notably, the two IPAs (CBS1 and CBS2) emerged as the most distinct beer styles, clearly separated in a three-dimensional space, as depicted in the Figure. Conversely, CBS3 and CBS4 were discriminated against but exhibited proximity and occupied a similar region of space. To understand the variables contributing most to the observed patterns, a loading plot was applied. This graphical representation visually depicts the relationships between variables and principal components, facilitating the interpretation of complex datasets. Each polyphenolic compound is represented as a point in a scatter plot, its position determined by its correlation with the underlying components. Apigenin, brutieridin, and neoritrocin closely align with CBS1, showcasing a strong association with this beer (Figure 4). On the other hand, vanillic acid and caffeic acid distinctly discriminate CBS3 and CBS4. Bitter units, total phenols, and other polyphenolic compounds exhibit a robust association with CBS2 (Figure 4). Variables positioned farther away denote weaker relationships. Furthermore, the phenolic content in beer is contingent on the types of barley and hops used in production. Despite hops containing a substantial amount of phenols (up to 4% of dry matter) compared to barley (up to 0.1%), it is noteworthy that, on average, four-fifths of the phenols in beer originate from malt or other mashed cereals due to their significantly higher initial content. Our findings revealed variations linked to the diverse malts and cereals employed, indicating that the presence of bergamot peels had no discernible impact on phenolic compound expression. However, it did influence the flavonoid content. Employing statistical methods, we not only discerned variations among beer styles but also traced the compounds responsible for distinguishing these typologies. This not only contributes to quality assurance for confirming beer styles but also advances our understanding of the intricate chemistry influencing beer quality.

## 5. Conclusions

In this study, a UHPLC-PDA method was developed and validated for the simultaneous determination of hop polyphenols and bitter acids in beer samples, using a direct sample injection system. The method developed allowed us to determine numerous polyphenols with excellent chromatographic separations and to quantify typical phenols of beers, such as p-Coumaric acid, 4-Hydroxybenzoic acid, and Protocatechuic acid, and also compounds derived from “characterizing foods” such as neoeriocitrin, naringin, and neohesperidin. The method made it possible to identify numerous classes of bitter acids such as lupolones, humulones, and the corresponding isomeric forms. The UHPLC system with the sub-2 core–shell column allowed us to separate compounds with very similar structures such as cis–trans pairs. The method was applied to four different beers, and the qualitative results for the bitter acids and the quantitative results for the polyphenols are in line with the data in the literature. The application of a streamlined analytical protocol, requiring equipment that offers extensive information on beer chemistry but remains more accessible than LC-MS in terms of cost and complexity, holds tremendous potential. The ease and speed of analysis with a non-expensive instrument make this method great for many beer supply chain applications. The validated method can be used by breweries to carry out a kidnapped screening of polyphenols and bitter acids of a beer in the production phase to perfect taste, flavor, and style or to improve production techniques and use of raw materials. Furthermore, the method described can be a powerful tool to determine food fraud (use of non-natural essences) and to determine the conservation status of beers.

## Data Availability

The original contributions presented in the study are included in the article, further inquiries can be directed to the corresponding author.

## Supplementary Materials

The following supporting information can be downloaded at: https://www.mdpi.com/article/10.3390/foods13081149/s1.
