# Supplementary material for: UHPLC-PAD Protocol for the Simultaneous Identification of Polyphenols and Bitter Acids: Organoleptic and Nutraceutical Fingerprinting of Bergamot-Flavored Craft Beer
Source: Foods. 2024 Apr 10;13(8):1149. doi: 10.3390/foods13081149 (PMC11049281; doi:10.3390/foods13081149)
Supplement: Supplementary file 1 [file foods-13-01149-s001.zip › foods-2929991-supplementary.pdf]

Table S1 Validation parameters values determined for the polyphenols.

Compounds	Linear range (ppm)	Regression equation	R^2	LOD (ppm)	LOQ (ppm)	Repeatbility (CV %)	Reproducibility (CV %)
Gallic acid	0.5-100	y = 58838.4809x – 34304.6944	0.9996	0.065	0.218	5.16	5.19
Protocatechuic acid	0.5-100	y = 56968.3550x + 10041.8594	0.9996	0.079	0.264	5.57	4.19
4-Hydroxybenzoic acid	0.5-100	y = 66976.5781x + 8811.2215	0.9996	0.047	0.156	3.46	3.42
Vanillic acid	0.5-100	y = 39046.0740x – 17589.1592	0.9998	0.076	0.254	5.69	6.00
Caffeic acid	0.5-100	y = 80.403.9809x – 5.618.2263	0.9998	0.050	0.167	3.892	2.89
Chlorogenic acid	0.75-120	y = 37088x – 53084	0.9992	0.084	0.281	5.08	5.34
Syrengin acid	0.5-100	y = 45154.2555x – 19290.0310	0.9998	0.081	0.270	5.81	5.01
Ethylgallate	0.5-100	y = 43307.1523x + 2133.0116	0.9997	0.075	0.250	5.68	4.30
(-)Epicatechin	0.7-110	y = 8995.0663x – 8543.3500	0.9993	0.075	0.251	4.90	4.05
Ferulic acid	0.5-100	y = 76190.7484x – 15485.2230	0.9999	0.062	0.205	4.72	3.50
Coumaric acid	0.5-100	y = 101919.0398x - 19710.9815	0.9998	0.033	0.097	1.81	1.49
Eriocetrin	0.5-100	y = 22104.7983x – 5509.6099	0.9997	0.040	0.096	1.39	4.19
Neoeriocitrin	0.5-100	y = 21193.8726x – 8292.7441	0.9998	0.043	0.086	1.66	2.13
Rutin	0.7-110	y = 26417.8491x – 27143.8067	0.9993	0.048	0.159	3.40	3.79
Narirutin	0.5-100	y = 22293x + 2686.4	0.9995	0.106	0.355	4.78	5.53
Naringin	0.7-110	y = 23180.1541x – 18258.1645	0.9994	0.034	0.081	1.71	6.00
Myricetin	0.5-100	y = 44789.0025x – 37079.9968	0.9997	0.057	0.190	6.38	5.41
Hesperidin	0.5-100	y = 61439.7309x – 25498.1206	0.9998	0.053	0.175	1.24	2.56
Diosmin	0.5-100	y = 4214.8056x + 1093.5404	0.9987	0.077	0.255	1.19	1.24
Neohesperidin	0.5-100	y = 10709.8118x + 3447.8446	0.9993	0.032	0.106	1.36	1.65
Neodiosmin	0.5-100	y = 22188.5733x – 11478.9917	0.9996	0.039	0.083	1.45	4.38
Naringenin	0.5-100	y = 36851.3110x – 17639.1841	0.9997	0.033	0.077	1.69	3.69
Apigenin	0.5-100	y = 68168.9349x – 21240.6742	0.9994	0.036	0.120	2.81	3.23
Kampferol	0.5-100	y = 35708.4046x – 8738.4609	0.9997	0.051	0.171	5.41	3.82
Hesperetin	0.5-100	y = 25158.0962x – 8615.2595	0.9999	0.040	0.107	1.22	2.39
Isorhamnetin	0.5-100	y = 44346.4593x – 26794.1428	0.9997	0.028	0.093	2.45	3.98
Rhamnetin	0.5-100	y = 41523x – 22496	0.9995	0.048	0.159	5.10	4.15
Sinensetin	0.5-100	y = 52911.3069x – 17330.2806	0.9995	0.029	0.076	1.01	0.99
Nobiletin	0.5-100	y = 48288.0608x – 24771.5556	0.9995	0.038	0.078	1.21	2.52
Tangeretin	0.5-100	y = 63271x – 8409.3	0.9995	0.050	0.110	0.67	2.02

Table S2 Retention time, instrumental recovery and percentage relative standard deviation of polyphenols

Compounds	±SD	RSD%	Recovery (%)	Compounds	±SD	RSD%	Recovery (%)
Coumaric acid	0.008	0.496	102.1	Neoeriocitrin	0.011	0.772	95.1
4-Hydroxybenzoic acid	0.014	1.310	103.8	Hesperetin	0.018	0.246	96.0
Caffeic acid	0.011	0.601	102.0	(-)Epicatechin	0.013	0.868	93.9
Ethylgallate	0.020	1.396	102.7	Eriocetrin	0.015	0.657	97.0
Ferulic acid	0.007	0.258	101.9	Isorhamnetin	0.019	1.357	95.6
Gallic acid	0.012	0.208	94.6	Myricetin	0.019	1.066	95.0
Kampferol	0.014	0.767	100.8	Neohesperidin	0.042	0.604	100.8

Naringin	0.010	0.358	93.2	Diosmin	0.011	2.077	86.9
Protocatechuic acid	0.009	0.769	102.3	Narirutin	0.016	0.931	105.1
Rutin	0.012	0.668	93.2	Rhamnetin	0.008	0.368	102.3
Syrengin acid	0.008	0.283	97.3	Tangeretin	0.019	0.964	104.4
Vanillic acid	0.015	1.172	95.9	Apigenin	0.009	0.655	102.8
Hesperidin	0.007	0.321	95.8	Chlorogenic acid	0.015	0.837	95.9
Sinensetin	0.006	0.385	108.2	Nobiletin	0.006	0.077	99.7
Neodiosmin	0.014	0.356	95.5	Naringenin	0.011	0.101	99.6

Table S3 Separation scheme of the bitter acids contained in the analyzed standards

Peak	ICE-4	ICS-T3	ICS-H2	ICS-I4	ICS-R3	Rt
1				trans-Isohumulone		25.044
2		trans-Tetrahydroiso, Cohumulone	cis-Hexahydroiso cohumulone (1)	trans-Isocohumulone	cis-ϱ-Isocohumulone	25.119
3					cis-ϱ-Isohumulone	25.715
4	Adlupulone, Lupulone					25.853
5		cis-Tetrahydroiso, Cohumulone				26.175
6				trans-Isoadhumulone	cis-ϱ-Isoadhumulone	26.380
7	Cohumulone		cis-Hexahydroiso cohumulone (2)			26.489
8			cis-Hexahydroiso Humulone			26.752
9		trans-Tetrahydroiso, Humulone				27.205
10			cis-Hexahydroiso adhumulone			27.491
11		cis-Tetrahydroiso, Humulone				27.596
12	Adhumulone, Humulone					27.625
13	Colupulone					27.813
14		cis-trans-Tetrahydro isoadhumulone				28.302

Table S4 Equation of calibration curves in solvent and in matrix and matrix effect value (ME%)

	**Equation Standard**	**Blond beer equation**	**Dark beer equation**	**ME% Blond**	**ME% Dark**
**4-Hydroxybenzoic acid**	y = 66.976.58x + 8.897.48	y = 60344.72x + 122.895.95	y = 56794.11x + 2.832.079.45	90.1%	84.8%
**Tangeretin**	y = 63.271.42x - 8.409.31	y = 57862.28x - 81.582.57	y = 52817.02x - 45.756.09	91.5%	83.5%

Table S5 Retention time of polyphenols and ICE-4 standard containing α-β-acids

N	Compounds	Rt	N	Compounds	Rt
1	Gallic acid	1.07	18	Hesperidin	14.531
2	Protocatechuic acid	2.157	19	Diosmin	14.864
3	4-Hydroxybenzoic acid	4.06	20	Neohesperidin	14.925
4	Vanillic acid	7.344	21	Neodiosmin	15.191
5	Caffeic acid	7.751	22	Naringenin	17.644
6	Chlorogenic acid	8.637	23	Apigenin	18.489
7	Syrengin acid	8.996	24	Kampferol	18.551
8	Ethylgallate	9.836	25	Hesperetin	18.558
9	(-)Epicatechin	9.88	26	Isorhamnetin	19.087
10	Ferulic acid	11.323	27	Rhamnetin	20.826
11	Coumaric acid	12.396	28	Sinensetin	21.488
12	Eriocetrin	12.597	29	Nobiletin	22.432
13	Neoeriocitrin	12.986	30	Tangeretin	23.29
14	Rutin	13.01	31	Adlupulone+ Lupulone	25.853
15	Narirutin	13.721	32	Cohumulone	26.489
16	Naringin	14.125	33	Adhumulone+ Humulone	27.625
17	Myricetin	14.332	34	Colupulone	27.813
